# Associations of neighborhood sociodemographic environment with mortality and circulating metabolites among low-income black and white adults living in the southeastern United States

**DOI:** 10.1186/s12916-024-03452-6

**Published:** 2024-06-18

**Authors:** Kui Deng, Meng Xu, Melis Sahinoz, Qiuyin Cai, Martha J. Shrubsole, Loren Lipworth, Deepak K. Gupta, Debra D. Dixon, Wei Zheng, Ravi Shah, Danxia Yu

**Affiliations:** 1 Division of Epidemiology, Department of Medicine, Vanderbilt Epidemiology Center, Vanderbilt University Medical Center, 2525 West End Avenue, Nashville, TN 37203 USA; 2 Department of Biostatistics, Vanderbilt University Medical Center, Nashville, TN USA; 3 Division of Cardiovascular Medicine, Department of Medicine, Vanderbilt Translational and Clinical Cardiovascular Research Center, Vanderbilt University Medical Center, Nashville, TN USA; 4 International Epidemiology Field Station, Vanderbilt University Medical Center, Nashville, TN USA

**Keywords:** Neighborhood deprivation, Residential segregation, Social vulnerability, Mortality, Metabolomics

## Abstract

**Background:**

Residing in a disadvantaged neighborhood has been linked to increased mortality. However, the impact of residential segregation and social vulnerability on cause-specific mortality is understudied. Additionally, the circulating metabolic correlates of neighborhood sociodemographic environment remain unexplored. Therefore, we examined multiple neighborhood sociodemographic metrics, i.e., neighborhood deprivation index (NDI), residential segregation index (RSI), and social vulnerability index (SVI), with all-cause and cardiovascular disease (CVD) and cancer-specific mortality and circulating metabolites in the Southern Community Cohort Study (SCCS).

**Methods:**

The SCCS is a prospective cohort of primarily low-income adults aged 40–79, enrolled from the southeastern United States during 2002–2009. This analysis included self-reported Black/African American or non-Hispanic White participants and excluded those who died or were lost to follow-up ≤ 1 year. Untargeted metabolite profiling was performed using baseline plasma samples in a subset of SCCS participants.

**Results:**

Among 79,631 participants, 23,356 deaths (7214 from CVD and 5394 from cancer) were documented over a median 15-year follow-up. Higher NDI, RSI, and SVI were associated with increased all-cause, CVD, and cancer mortality, independent of standard clinical and sociodemographic risk factors and consistent between racial groups (standardized HRs among all participants were 1.07 to 1.20 in age/sex/race-adjusted model and 1.04 to 1.08 after comprehensive adjustment; all *P* < 0.05/3 except for cancer mortality after comprehensive adjustment). The standard risk factors explained < 40% of the variations in NDI/RSI/SVI and mediated < 70% of their associations with mortality. Among 1110 circulating metabolites measured in 1688 participants, 134 and 27 metabolites were associated with NDI and RSI (all FDR < 0.05) and mediated 61.7% and 21.2% of the NDI/RSI-mortality association, respectively. Adding those metabolites to standard risk factors increased the mediation proportion from 38.4 to 87.9% and 25.8 to 42.6% for the NDI/RSI-mortality association, respectively.

**Conclusions:**

Among low-income Black/African American adults and non-Hispanic White adults living in the southeastern United States, a disadvantaged neighborhood sociodemographic environment was associated with increased all-cause and CVD and cancer-specific mortality beyond standard risk factors. Circulating metabolites may unveil biological pathways underlying the health effect of neighborhood sociodemographic environment. More public health efforts should be devoted to reducing neighborhood environment-related health disparities, especially for low-income individuals.

**Supplementary Information:**

The online version contains supplementary material available at 10.1186/s12916-024-03452-6.

## Background

Although overall mortality rates and those attributable to cardiovascular disease (CVD) and cancer steadily declined in the United States (US) between 1990 and 2019, significant health disparities persist by race/ethnicity, socioeconomic status (SES), and geographic region, with higher mortality rates among Black or African American adults compared with non-Hispanic White adults [1–3]. Society and community-level factors are fundamental drivers of health disparities [3–5]. Residing in an area with greater socioeconomic deprivation, marginalization, or vulnerability has been associated with increased mortality, regardless of individual-level SES and health risk factors. For example, in the Southern Community Cohort Study (SCCS)—a prospective cohort of ~ 84,000 adults recruited from 12 southeast states in the US, mostly with low SES and two-thirds being self-identified Black/African American, we found a ~ 10–25% increased all-cause mortality for the highest vs. lowest quartiles of a neighborhood deprivation index (NDI), even with adjustment for individual SES and lifestyle factors [6, 7]. Consistent results have been reported from other large US cohorts [8–13], with the SCCS being a unique racially diverse cohort of mostly low-SES individuals with major risk factors available for adequate model adjustment and biological samples for mechanistic investigation.

The potential pathways linking neighborhood sociodemographic disadvantages with increased mortality are physical, social, and biological, involving the built environment, health care access and quality, social cohesion, individual health-related behaviors, and underlying biological changes [14, 15]. Disadvantaged and marginalized neighborhoods are more likely to be exposed to environmental pollution, violence, and discrimination while having limited access to financial resources, exercise facilities, nutritious foods, and health care [16–19]. Those exposures have been linked to multiple mortality risk factors, including obesity, smoking, poor-quality diet, mental health disorders (e.g., anxiety, depression), metabolic disorders (e.g., high blood pressure, blood lipids, diabetes, chronic inflammation), and CVD [15, 20–24]. Despite convincing evidence on associations of neighborhood disadvantage with diseases and mortality, the complex biological and metabolic profiles correlated with neighborhood environments remain largely unknown [25], particularly among low-SES Black adults. Studies investigating neighborhood environments with circulating biomarkers were conducted predominantly among White adults or middle-to-high SES individuals [22, 26–29]. Given that low-SES individuals may particularly rely on neighborhood resources while suffering from persistent social inequalities and health disparities, research among racially/ethnically diverse, low-SES individuals is critical for developing interventions and policies to reduce health disparities systematically.

In this study, we hypothesized that neighborhood sociodemographic environment, measured by the residential segregation index (RSI, i.e., Index of Concentration at the Extremes [ICE] [30], a surrogate marker of structural racism) and social vulnerability index [31, 32] (SVI, a measure of community resilience if confronted by disasters), as well as NDI, was associated with all-cause and CVD and cancer-specific mortality. Routinely considered individual SES and clinical risk factors may partially explain and mediate the association between neighborhood sociodemographic environment and mortality, but not entirely. In addition, given the central role of metabolism in human health and health disparities [33, 34], we hypothesized that plasma metabolomics could offer potential novel insights into biological pathways underlying the influence of neighborhood sociodemographic environment on human health.

## Methods

### Study population

This study was based on the SCCS, which was approved by the Institutional Review Boards of the Vanderbilt University Medical Center and Meharry Medical College, with written informed consent obtained from all SCCS participants. Detailed cohort design and protocol of SCCS were described previously [35]. In brief, 84,069 participants, aged 40–79 years, were recruited from 12 southeastern US states during 2002–2009. Most participants (≈86%) were recruited from community health centers that served low-income, medically uninsured/underinsured individuals (~ 65% were self-reported Black/African American, and > 50% had annual household income < $15,000). At baseline, demographics, lifestyles, and medical history were obtained using structured questionnaires, including age, sex, race/ethnicity, educational attainment, household income, insurance coverage, tobacco smoking, alcohol drinking, physical activity—by total metabolic equivalent hours per day [36, 37], sleep hours, habitual food intakes and overall diet quality—by Healthy Eating Index-2010 [38], body mass index (BMI), depression score—by the Center for Epidemiologic Studies Depression Scale (CESD-10) [39], and histories of diabetes, hypertension, chronic obstructive pulmonary disease (COPD), CVD, and cancer. Participants were followed via surveys and linkages to disease and death registries.

For the present study, we only included self-reported Black/African American or non-Hispanic White participants, given the small number of participants in other racial/ethnic groups (*n* = 4098 were excluded). We also excluded participants who died or were lost to follow-up within 1 year (*n* = 340 were excluded), leaving 79,631 individuals in our primary analysis with mortality. Among them, all participants had at least one measure of NDI, RSI, and SVI, and missing data in exposure variables or covariates were handled by complete-case analysis (i.e., participants with missing data were excluded from the relevant model). A subset of 1688 SCCS participants were included in the analysis with circulating metabolites. They were selected in nested case–control studies of incident coronary heart disease (CHD) (*n* = 1023) [40] and incident prostate cancer (*n* = 665). For both nested case–control studies, the included participants had no history of cancer and provided plasma samples at baseline. For the CHD study, participants also had (1) no history of CHD, stroke, heart failure, or end-stage renal disease at baseline; (2) data on fasting time and time between sample collection and lab processing; (3) no use of antibiotics nor cold/flu in last 7 days before blood collection (to avoid the influence of recent infection); and (4) eligibility for Centers for Medicare and Medicaid Services and at least 2 claims after SCCS enrollment (to facilitate CHD case identification).

### Measures of neighborhood sociodemographic environment

We constructed three well-described metrics reflecting neighborhood deprivation (NDI), residential segregation (RSI), and social vulnerability (SVI)—via geocoding and linkage to the US 2000 Census data [7, 41, 42].

Specifically, NDI was constructed through principal component analysis using 11 census tract-level variables [7, 42, 43], representing four dimensions: social indicators (percentage of occupied housing units with renter/owner costs > 50% of income; percentage of housing units with ≥ 1 occupant per room; percent female-headed households with dependent children), wealth and income (percentage of persons with income below the 1999 poverty status; percentage of households with income < $30,000 per year; percentage of households with public assistance income; percentage of households with no car; median value of owner-occupied housing units), education (percentage of persons aged ≥ 25 years that did not graduate high school), and occupation (percentage of males and females who are unemployed; percent males in professional occupations). The first principal component was retained for NDI construction, which explained > 60% of the variability [7]. Higher values of NDI represent greater neighborhood deprivation.

RSI measures residential segregation and is considered a surrogate marker of structural racism, quantified by a generalized formula of ICE per Krieger and colleagues [44–46]. A census tract-level ICE was constructed by calculating the difference between the number of individuals belonging to a deprived extreme (defined as low-income Black) and the number of individuals belonging to a privileged extreme (defined as high-income White) indexed to the number of individuals living in that census tract [30]; thus, the range of ICE is − 1 to + 1. Higher values represent higher concentrations of low-income Black individuals in an area. ICE has been linked to BMI, hypertension, mental disorders, and mortality with potential differences in the association by race [30, 46–48]. We used the ICE data from the National Cancer Institute Social Determinants of Health, derived from 2008–2012 American Community Survey 5-Year estimates.

SVI measures a community’s ability to prevent human suffering and financial loss in the event of a disaster (e.g., disease outbreaks or natural disasters). SVI is based on 16 census tract-level variables that represent four themes: SES (below 150% poverty, unemployed, housing cost burden, no high school diploma, and no health insurance), household characteristics (aged 65 and older, aged 17 and younger, civilian with a disability, single-parent households, and English language proficiency), racial/ethnic minority status, and housing type/transportation (multi-unit structures, mobile homes, crowding, no vehicle, and group quarters) [31, 32]. It is an overall ranking from the ranking of each constituted tract-level variable and ranges from 0 to 1. Higher SVI values denote greater vulnerability. We used SVI data from the Centers for Disease Control and Prevention’s Agency for Toxic Substances and Disease Registry [49].

### Mortality ascertainment

Vital status and the date and underlying cause of death were obtained via linkages to the National Death Index and Social Security Administration vital status service for epidemiologic research through December 31, 2020. Deaths due to CVD (I00–I78) and cancer (C00–C97) were ascertained by ICD-10 codes.

### Plasma metabolite profiling

Plasma metabolite profiling was conducted in a subset of SCCS participants (*n* = 1688; see “Study population”). Ultra-high-performance liquid chromatography coupled with tandem mass spectrometry was performed by Metabolon, Inc. (Morrisville, NC) [40, 50], which detected ~ 1500 metabolites with 1229 known metabolites. After excluding metabolites missing in > 20% of participants, 1110 metabolites were used for analysis. Other missing metabolites were imputed by half the minimal values of non-missing data [51–53]. Metabolite levels were log-transformed and standardized to *Z*-scores (mean 0 and unit variance) before analysis.

### Statistical analysis

#### Neighborhood sociodemographic environment with total and cause-specific mortality

We first examined the distributions of NDI, RSI, and SVI and their Spearman correlations to understand the joint variation of these metrics in our population. Then, to estimate whether neighborhood sociodemographic environment was associated with leading causes of mortality, independent of standard sociodemographic and clinical factors, we used Cox regression to evaluate the relation of each metric (scaled to unit variance) with all-cause mortality across three models, adjusting for age, sex, and self-reported race (model 1); additional adjustment for individual-level SES (education, annual household income, and insurance coverage) (model 2); and additional adjustment for lifestyle and health factors (smoking status, alcohol drinking, physical activity, diet quality, sleep hours, BMI, depression score, and history of diabetes, hypertension, COPD, CVD, and cancer) (model 3). Participants with missing data in any exposure variable or covariates were excluded from the relevant model (for NDI: *n* = 0, 2650, and 11,738 were removed from models 1–3, respectively; for RSI, *n* = 6251, 8754, and 17,047 were removed from models 1–3, respectively; for SVI, *n* = 7, 2657, and 11,745 were removed from models 1–3, respectively; characteristics of participants included in analysis and those excluded due to missingness were shown in Additional file 2). Violation of proportional hazards assumption (assessed via Schoenfeld residual) was handled via stratification or time-dependent covariate adjustment. Continuous covariates were included as restricted cubic splines to account for non-linear relations, while NDI, RSI, and SVI all showed linear associations with mortality. We used a competing risk framework within Cox regression (Fine-Gray subdistribution hazard model) to examine the relation of each neighborhood metric with CVD or cancer-specific mortality. Models were performed among all participants and stratified by self-reported race, age (< median and ≥ median), or sex. *P* for interaction was assessed by adding an interaction term (neighborhood variable × self-reported race, age, or sex) to the regression model. Given three neighborhood metrics were evaluated, we applied Bonferroni correction and considered two-sided *P* < 0.016 (0.05/3) statistically significant.

We also used multivariable regression to estimate the variances (adjusted *R*^2^) in NDI, RSI, and SVI captured by standard SES measures and clinical risk factors (i.e., routinely considered individual-level socioeconomic, lifestyle, and health factors, including education, annual household income, insurance coverage, smoking status, alcohol drinking, physical activity, diet quality, sleep hours, BMI, depression score, and histories of diabetes, hypertension, COPD, CVD, and cancer). We used multiple mediation analysis [54] to evaluate the potential mediation effect of those standard risk factors on the relation of neighborhood sociodemographic environment with mortality (age, sex, and self-reported race adjusted). Such analyses were conducted among all participants and by self-reported race.

#### Identifying metabolite correlates of neighborhood sociodemographic environment

Linear regression was used to identify plasma metabolites associated with NDI, RSI, and SVI, adjusting for age, sex, self-reported race, fasting status, and assay batch. A two-sided Benjamini–Hochberg false discovery rate (FDR) < 0.05 was considered statistically significant. Then, metabolite signatures of NDI/RSI/SVI were constructed by summing the *Z*-scores of significant metabolites using an unweighted method and considering the direction of the association. Mediation analysis was used to evaluate the mediation effects of metabolite signatures on the associations between neighborhood metrics and all-cause mortality. We also assessed the mediation effect of standard risk factors on the neighborhood environment-mortality association and compared the mediated proportions after adding the metabolite signatures. Finally, we performed pathway enrichment analysis to identify metabolite sub-pathways, using the hypergeometric test similar to previous studies [55–57], with 120 sub-pathways and 1229 known metabolites as background pathways and metabolite sets, respectively. All statistical analyses were conducted in R (version: 4.1.1).

## Results

### Baseline characteristics

**Fig. 1:**
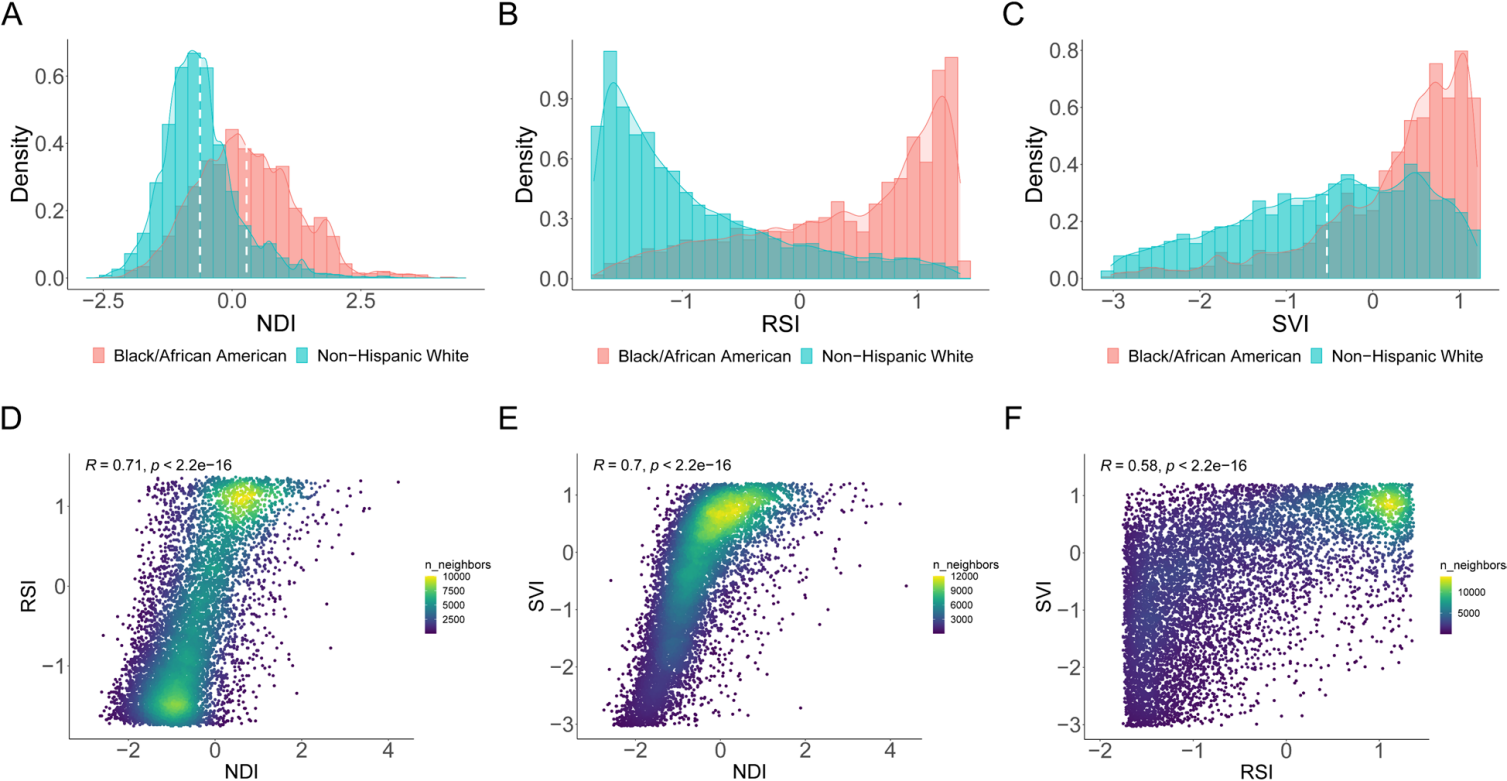
The distributions of neighborhood sociodemographic environment metrics between self-reported Black/African American and non-Hispanic White participants and their mutual correlations in the Southern Community Cohort Study. The distributions of **A** NDI, **B** RSI, and **C** SVI between self-reported Black/African American and non-Hispanic White participants. Spearman correlations between **D** NDI and RSI, **E** NDI and SVI, and **F** RSI and SVI. NDI, neighborhood deprivation index; RSI, residential segregation index; SVI, social vulnerability index

Among 79,631 study participants, the median age was 51 years (interquartile range [IQR]: 45–58); 59.6% were female (*N* = 47,436), and 68.7% were self-reported Black/African American (*N* = 54,734; Table 1). For both self-reported Black/African American and non-Hispanic White participants, those who survived generally exhibited a lower NDI, SVI, and RSI, lower rates of chronic illness, better lifestyle measures, and higher education and income levels (Table 1). Even though most SCCS participants had low SES regardless of self-reported race, Black/African American participants had evidence of neighborhood deprivation, segregation, and vulnerability, as shown by higher median levels of NDI, RSI, and SVI (Fig. 1A–C). These variables were highly intercorrelated (Fig. 1D–F), reflecting complex interplay in neighborhood sociodemographic ecology.

**Table 1:** Baseline characteristics of study participants

	**No./total no. (%)**				
	Total participants (*N*= 79,631)	**Self-reported Black/African American participants**		**Self-reported non-Hispanic White participants**	
		Alive (*N* = 38,991)	Death (*N* = 15,743)	Alive (*N* = 17,284)	Death (*N* = 7613)
Age, median (IQR), y*	51 (45–58)	49 (44–55)	54 (48–61)	52 (46–59)	57 (49–64)
Sex					
Female	47,436/79,631 (59.6)	24,393/38,991 (62.6)	7767/15,743 (49.3)	11,222/17,284 (64.9)	4054/7613 (53.3)
Male	32,195/79,631 (40.4)	14,598/38,991 (37.4)	7976/15,743 (50.7)	6062/17,284 (64.9)	3559/7613 (46.7)
Education					
< High school	22,715/78,572 (28.9)	10,626/38,387 (27.7)	6299/15,469 (40.7)	3460/17,167 (20.2)	2330/7549 (30.9)
High school	45,703/78,572 (58.2)	23,451/38,387 (61.1)	8100/15,469 (52.4)	9841/17,167 (57.3)	4311/7549 (57.1)
College	10,154/78,572 (12.9)	4310/38,387 (11.2)	1070/15,469 (6.9)	3866/17,167 (22.5)	908/7549 (12.0)
Annual household income					
< $15,000	42,869/77,518 (55.3)	20,952/37,921 (55.3)	10,690/15,280 (70.0)	6718/16,872 (39.8)	4509/7445 (60.6)
$15,000–$24,999	16,374/77,518 (21.1)	8983/37,921 (23.7)	2883/15,280 (18.9)	3117/16,872 (18.5)	1391/7445 (18.7)
$25,000–$49,999	10,886/77,518 (14.0)	5487/37,921 (14.5)	1301/15,280 (8.5)	3159/16,872 (18.7)	939/7445 (12.6)
≥ $50,000	7389/77,518 (9.5)	2499/37,921 (6.6)	406/15,280 (2.7)	3878/16,872 (23.0)	606/7445 (8.1)
Insurance coverage	47,523/78,100 (60.8)	21,627/38,160 (56.7)	9940/15,370 (64.7)	10,976/17,068 (64.3)	4980/7502 (66.4)
Smoking status					
Current	31,852/78,014 (40.8)	15,202/38,152 (39.8)	7499/15,371 (48.8)	5549/17,004 (32.6)	3602/7487 (48.1)
Former	17,940/78,014 (23.0)	7423/38,152 (19.5)	3445/15,371 (22.4)	4870/17,004 (28.6)	2202/7487 (29.4)
Never	28,222/78,014 (36.2)	15,527/38,152 (40.7)	4427/15,371 (28.8)	6585/17,004 (38.7)	1683/7487 (22.5)
Alcohol drinking per day, median (IQR)*	0.02 (0–0.7)	0.02 (0–0.8)	0.02 (0–1.1)	0.01 (0–0.3)	0 (0–0.3)
Physical activity, median (IQR)*	9.7 (4.0–20.4)	11.1 (5.1–22.9)	8.0 (3.4–17.1)	10.3 (4.6–20.0)	8.0 (2.9–16.0)
Diet quality, median (IQR)*	57.4 (49.1–66.1)	57.6 (49.6–66.1)	57.3 (49.3–65.3)	57.6 (48.6–67.0)	56.1 (47.5–65.3)
Sleep hours, median (IQR)*	7.0 (6.0–8.0)	7.0 (6.0–8.0)	7.3 (6.0–8.0)	7.0 (6.0–8.0)	7.0 (6.0–8.0)
BMI, median (IQR)*	29.1 (24.9–34.4)	29.6 (25.4–34.9)	28.4 (24.1–34.2)	28.4 (24.7–33.5)	28.7 (24.4–34.2)
Depression score, median (IQR)*	8.0 (4.0–12.0)	8.0 (4.0–12.0)	8.0 (4.0–12.0)	7.0 (4.0–13.0)	9.0 (5.0–14.0)
History of diabetes	16,727/78,473 (21.3)	6617/38,350 (17.3)	5320/15,450 (34.4)	2544/17,139 (14.8)	2246/7534 (29.8)
History of hypertension	43,117/78,486 (54.9)	20,400/38,355 (53.2)	10,616/15,455 (68.7)	7522/17,140 (43.9)	4579/7536 (60.8)
History of COPD	6923/78,436 (8.8)	2374/38,338 (6.2)	1275/15,440 (8.3)	1749/17,123 (10.2)	1525/7535 (20.2)
History of CVD	9361/78,398 (11.9)	2916/38,314 (7.6)	2838/15,432 (18.4)	1716/17,128 (10.0)	1891/7524 (25.1)
History of cancer	6123/77,460 (7.9)	1502/37,914 (4.0)	978/15,333 (6.4)	2294/16,785 (13.7)	1349/7428 (18.2)
NDI, median (IQR)*	0.6 (− 0.2–1.5)	0.9 (0.2–1.8)	1.1 (0.4–2.0)	− 0.2 (− 0.6–0.3)	0 (− 0.5–0.6)
SVI, median (IQR)*	0.8 (0.6–0.9)	0.8 (0.7–0.9)	0.8 (0.7–0.9)	0.6 (0.4–0.8)	0.7 (0.5–0.8)
RSI, median (IQR)*	0.2 (− 0.5–0.7)	0.5 (− 0.0–0.8)	0.6 (0.1–0.8)	− 0.6 (− 0.9–[− 0.3])	− 0.6 (− 0.8–[− 0.1])

^*^The description of continuous variables, including age, alcohol drinking, physical activity, diet quality, sleep hours, BMI, depression score, NDI, SVI, and RSI, was based on 79,631, 77,208, 76,747, 73,648, 77,809, 77,675, 76,340, 79,631, 79,624, and 73,380 participants, respectively

### Neighborhood sociodemographic environment with total and cause-specific mortality

**Fig. 2:**
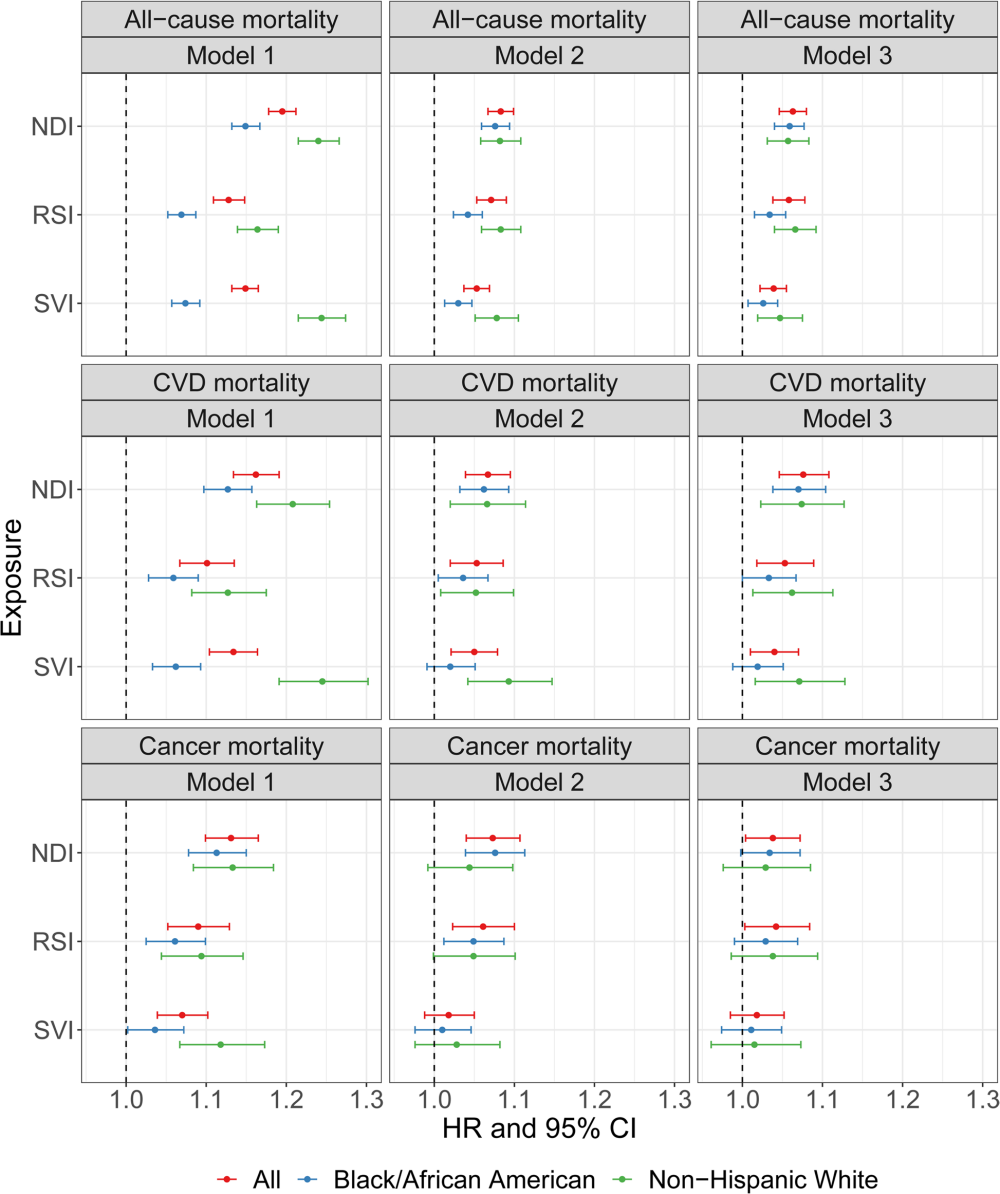
The associations of neighborhood sociodemographic environment metrics with all-cause and CVD and cancer-specific mortality in the Southern Community Cohort Study. Cox regression and competing risk framework within Cox regression were used to examine the associations of NDI, RSI, and SVI with all-cause mortality and CVD and cancer-specific mortality, respectively. Model 1 included age, sex, and self-reported race; model 2 additionally included education, annual household income, and insurance coverage; model 3 additionally included smoking status, alcohol drinking, physical activity, diet quality, sleep hours, BMI, depression score, and histories of diabetes, hypertension, COPD, CVD, and cancer. Models were performed among all participants and by self-reported race. NDI, neighborhood deprivation index; RSI, residential segregation index; SVI, social vulnerability index; CVD, cardiovascular disease; HR, hazard ratio; CI, confidence interval; COPD, chronic obstructive pulmonary disease

Over a median follow-up of 15 years (IQR: 12–16), we observed 23,356 deaths (7214 from CVD; 5394 from cancer). After adjusting for age, sex, and self-reported race, a higher NDI, RSI, or SVI was associated with higher all-cause mortality: hazard ratio (HR) and 95% confidence interval (CI) per standard deviation (SD) increase was 1.20 (1.18–1.21), 1.13 (1.11–1.15), and 1.15 (1.13–1.17), respectively (all *P* < 0.016 [0.05/3]; Fig. 2; Additional file 3). The associations were robust to further adjustment for individual-level socioeconomic, lifestyle, and health factors (models 2 and 3 in Fig. 2; Additional file 3). Point estimates of CVD and cancer-specific mortality were generally consistent with our findings for all-cause mortality, though associations with cancer mortality were generally mitigated (to *P* > 0.016) with more comprehensive adjustment.

**Fig. 3:**
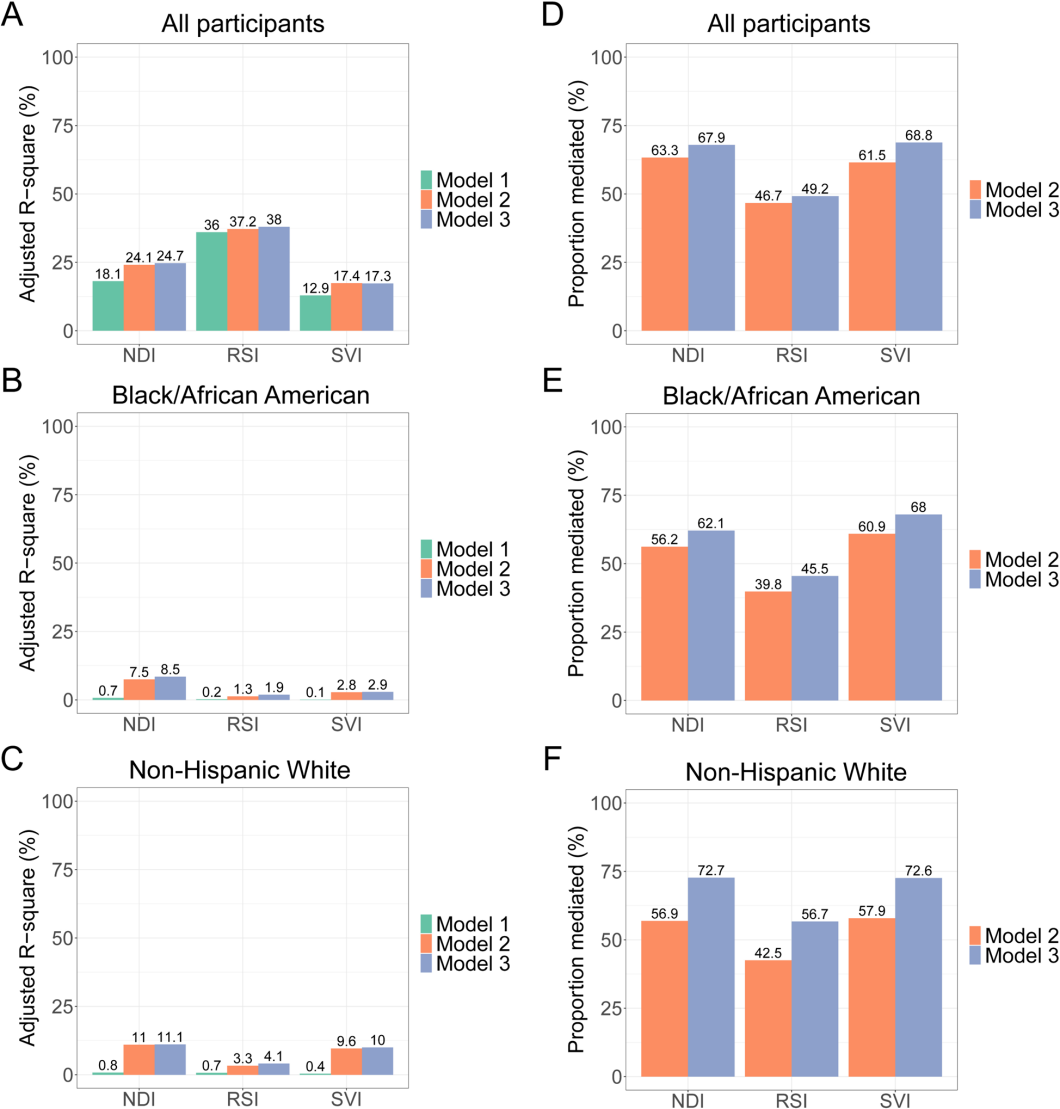
The variances in neighborhood sociodemographic environment metrics and their associations with mortality captured by standard SES and health risk factors. **A** The variance in NDI, RSI, and SVI captured by standard SES and health risk factors among all participants; **B** among self-reported Black/African American participants; **C** among self-reported non-Hispanic White participants. **D** The mediated variations of NDI/RSI/SVI-mortality association by standard SES and health risk factors among all participants; **E** among self-reported Black/African American participants; **F** among self-reported non-Hispanic White participants. Multivariable regression was used to estimate the variances in NDI, RSI, and SVI captured by standard SES and health risk factors. Multiple mediation analysis was used to evaluate the mediation effect of standard SES and health risk factors on the associations of NDI, RSI, and SVI with mortality. Model 1 included age, sex, and self-reported race; model 2 additionally included education, annual household income, and insurance coverage; model 3 additionally included smoking status, alcohol drinking, physical activity, diet quality, sleep hours, BMI, depression score, and histories of diabetes, hypertension, COPD, CVD, and cancer. Age, sex, and self-reported race were adjusted in the mediation analysis. NDI, neighborhood deprivation index; RSI, residential segregation index; SVI, social vulnerability index; SES, socioeconomic status; CVD, cardiovascular disease; COPD, chronic obstructive pulmonary disease

We observed larger HRs for all-cause and CVD-specific mortality in non-Hispanic White relative to Black/African American participants in model 1 (*P* for interaction < 0.016; Fig. 2; Additional file 3), but the differences became smaller upon comprehensive adjustment (most *P* for interaction > 0.016 in model 3). These results suggested apparent stronger associations of NDI, RSI, and SVI with mortality in non-Hispanic White participants may be confounded by variability in standard SES measures and risk factors (see “Methods”—“Statistical analysis”). This observation led us to evaluate (1) how much variations in NDI/RSI/SVI can be captured by standard risk factors and (2) what proportion of the associations between NDI/RSI/SVI and mortality may be mediated by standard risk factors. While higher NDI, RSI, and SVI were associated with poorer comorbidity, chronic illness, and individual-level SES, variances in NDI, RSI, or SVI could only be partially explained by these standard risk factors (adjusted *R*^2^ in model 3: 24.7%, 38%, and 17.3%, respectively; Fig. 3A). In turn, we observed variability in how standard risk factors mediated the relations of NDI, RSI, and SVI with mortality (67.9%, 49.2%, and 68.8%, respectively, for model 3; Fig. 3D). In addition, more variances in NDI, RSI, and SVI and their mortality associations were explained by standard risk factors among non-Hispanic White than Black/African American individuals (adjusted *R*^2^ in model 3: 11.1% vs. 8.5%, 4.1% vs. 1.9%, and 10% vs. 2.9% among non-Hispanic White and Black/African American individuals, for NDI, RSI, and SVI, respectively, Fig. 3B–C; proportion mediated in model 3: 72.7% vs. 62.1%, 56.7% vs. 45.5%, and 72.6% vs. 68% among non-Hispanic White and Black/African American individuals, for NDI, RSI, and SVI, respectively, Fig. 3E–F).

Similarly, we observed comparable associations of NDI, RSI, and SVI with mortality outcomes by age group or sex after comprehensive adjustment (most *P* for interaction > 0.016 in model 3; Additional file 1: Fig. S1 and Fig. S2; Additional file 4 and Additional file 5). However, stronger associations of NDI with all-cause and CVD mortality were found in women than in men (NDI with all-cause mortality in model 3: HR = 1.09 [1.07–1.12] vs. 1.04 [1.01–1.06], *P* for interaction < 0.001; NDI with CVD-specific mortality in model 3: HR = 1.11 [1.07–1.16] vs. 1.04 [1.00–1.08], *P* for interaction = 0.003; Additional file 1: Fig. S2 and Additional file 5).

### Neighborhood sociodemographic environment with circulating metabolites

**Fig. 4:**
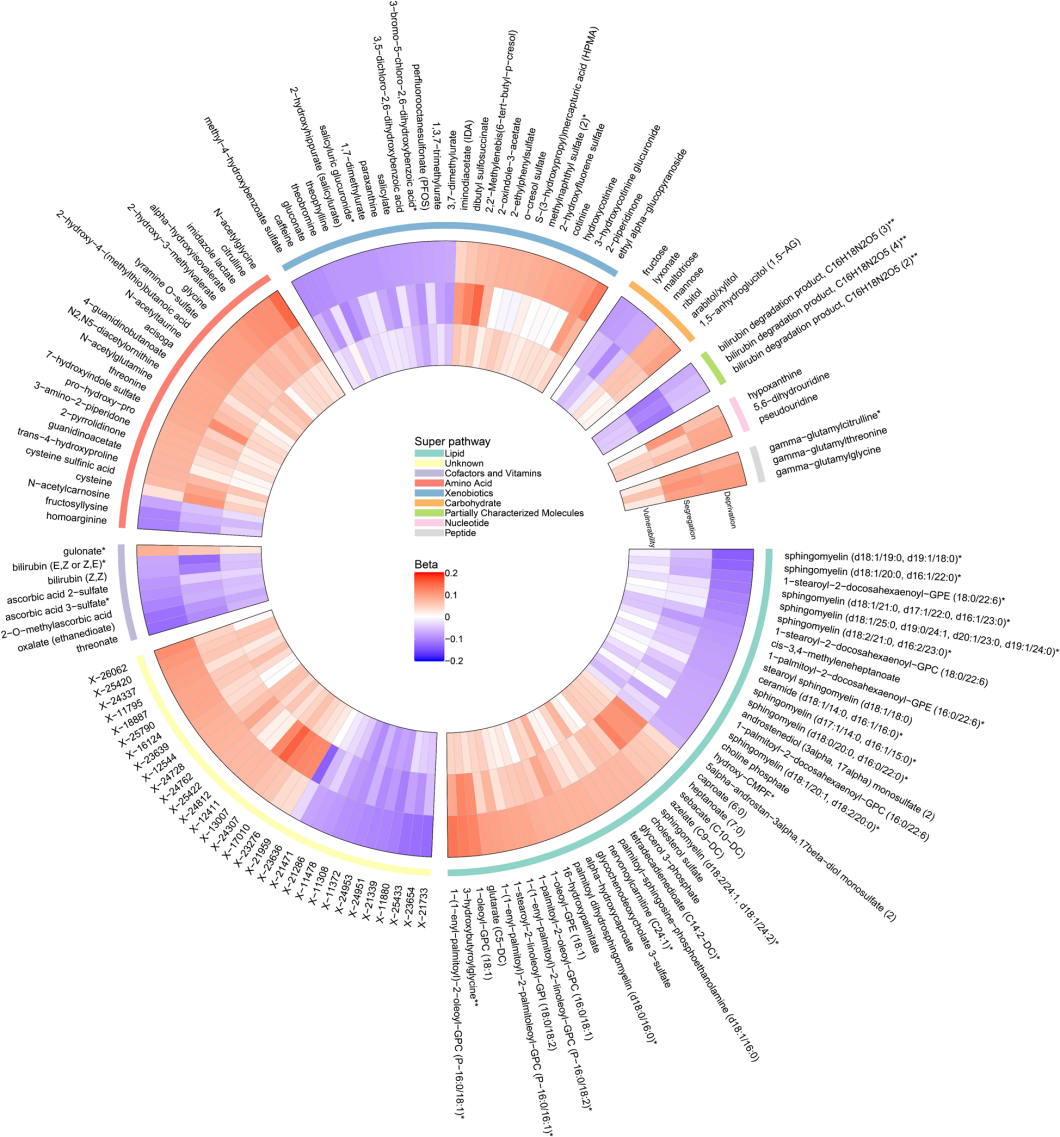
Circulating metabolites associated with neighborhood sociodemographic environment metrics. The associations were estimated by linear regression, adjusted for age, sex, self-reported race, fasting status, and batch. Metabolites associated with any neighborhood environment metrics at FDR < 0.05 were presented (a total of 152 metabolites)

**Fig. 5:**
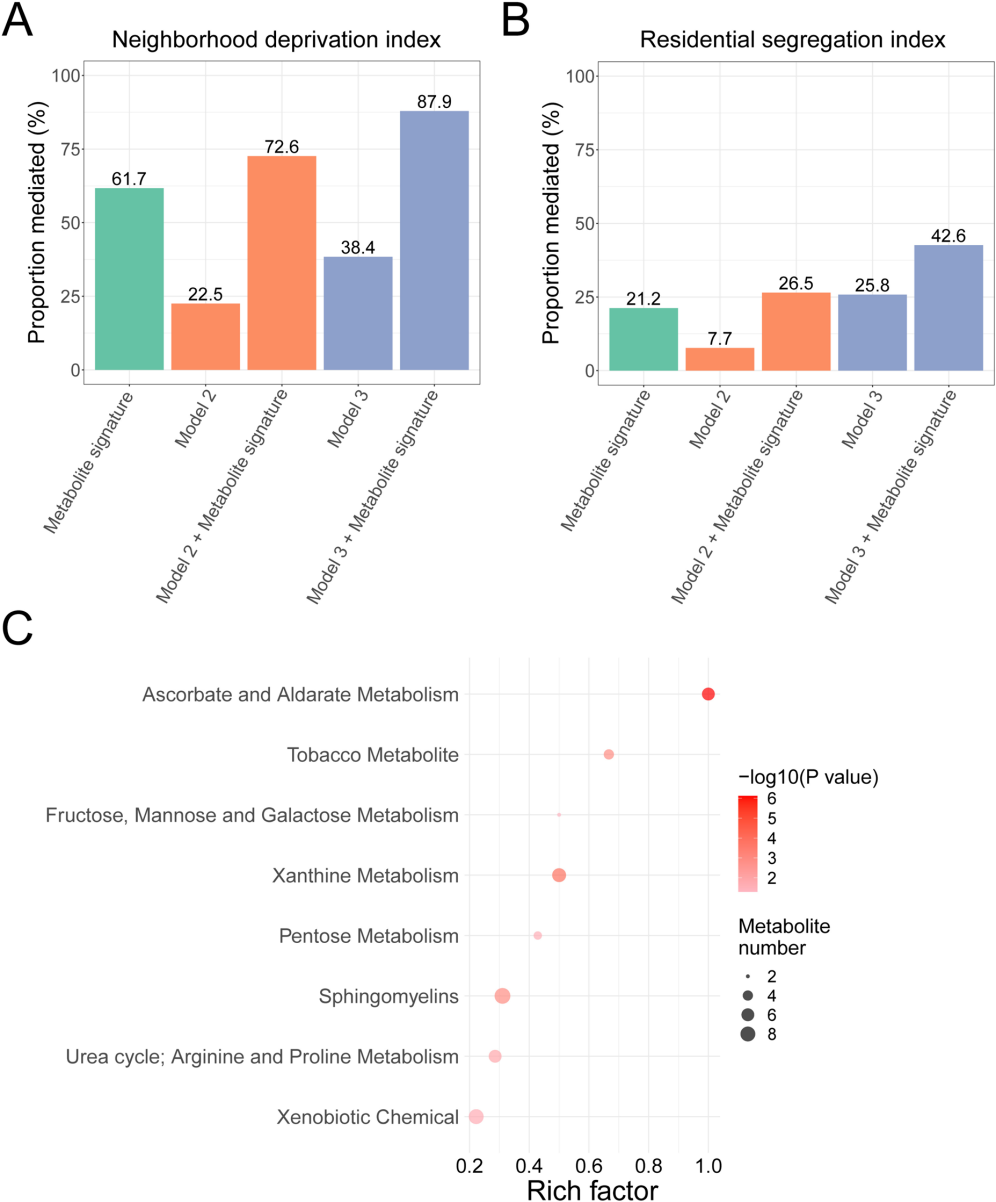
The mediation effects of metabolite signatures and standard risk factors on the association between neighborhood sociodemographic environment and all-cause mortality and the result of pathway enrichment analysis. **A** Mediation effects of metabolite signature, standard risk factors, and standard risk factors plus metabolite signature on the NDI-mortality association. **B** Mediation effects of metabolite signature, standard risk factors, and standard risk factors plus metabolite signature on the RSI-mortality association. In **A**–**B**, model 2 included age, sex, self-reported race, education, and annual household income; model 3 additionally included smoking status, alcohol drinking, physical activity, diet quality, sleep hours, BMI, depression score, and histories of diabetes, hypertension, COPD, CVD, and cancer. Age, sex, and self-reported race were adjusted in the mediation analysis. **C** Pathway enrichment analysis for 152 metabolites associated with NDI or RSI. *P* values were calculated by hypergeometric test. A total of 120 sub-pathways and 1229 known metabolites were used as background pathways and metabolite sets, respectively. Rich factor is the ratio of the number of selected metabolites to the number of all metabolites annotated in the sub-pathway. NDI, neighborhood deprivation index; RSI, residential segregation index; CVD, cardiovascular disease; COPD, chronic obstructive pulmonary disease

We identified 134 and 27 metabolites associated with NDI and RSI, respectively (at a 5% FDR, Additional file 1: Fig. S3 and Additional file 6), but none with SVI at 5% FDR. The directions of associations of identified metabolites were consistent across all metrics (Fig. 4). While most of these metabolites were annotated as lipids, amino acids, and xenobiotics, a significant fraction was unannotated (yellow color, Fig. 4). The metabolite signatures of NDI and RSI mediated 61.7% and 21.2% of their associations with all-cause mortality, respectively (Fig. 5A–B). In comparison, standard risk factors (as in model 3) explained 38.4% and 25.8% of the NDI/RSI-mortality association, respectively. When adding to standard risk factors, the metabolite signatures increased the mediation proportions to 87.9% and 42.6% for NDI and RSI, respectively (Fig. 5A–B). Pathway analysis showed eight enriched sub-pathways for metabolites associated with NDI or RSI (a total of 152 metabolites): ascorbate and aldarate metabolism, tobacco metabolite, fructose, mannose and galactose metabolism, xanthine metabolism, pentose metabolism, sphingomyelins, arginine and proline metabolism, and xenobiotic chemical pathways (*P* < 0.05; Fig. 5C and Additional file 7).

## Discussion

To our knowledge, this is the first study to examine residential segregation and social vulnerability with leading causes of mortality in a large cohort of 79,631 low-income adults living in the southeastern US and explore novel metabolite correlates/mediators of neighborhood sociodemographic environment. We showed that disadvantaged neighborhood sociodemographic environment (measured by NDI, RSI, and SVI) was associated with higher all-cause and cause-specific (CVD, cancer) mortality, independent of routinely considered clinical and sociodemographic risk factors. Moreover, we demonstrated that the standard risk factors account for < 40% of NDI/RSI/SVI variations and < 70% of their associations with mortality. In this context, we identified circulating metabolites associated with NDI and RSI, which may mediate the NDI/RSI-mortality association beyond standard risk factors and uncover potential metabolic pathways linking social determinants to human health. Our results suggest that markers of neighborhood-level deprivation, vulnerability, and segregation are critical factors in conveying mortality risk not fully explained by standard SES and clinical risk measures.

These results arise in a context of burgeoning evidence linking neighborhood sociodemographic disadvantage with increased mortality in US populations [8–13], though few studies have examined their role in individuals with low SES—a group at persistent higher mortality and systemic-structural disadvantages relative to middle-class Americans. Our study extended our prior studies in the SCCS by doubling the follow-up time and the number of CVD and cancer deaths and further demonstrated that higher NDI was significantly associated with greater CVD and cancer mortality, which could not be drawn in our previous studies due to relatively small numbers of CVD and cancer deaths at that time [6, 7]. In addition, our study, for the first time, examined the associations of residential segregation and social vulnerability with mortality among low-income Black/African American adults and non-Hispanic White adults; our results were consistent with previous studies conducted in other US populations [11, 58–60]. Of note, we found that higher residential segregation (measured by RSI), indicative of a higher percentage of low-income Black individuals in an area, was associated with higher mortality for both Black/African American and non-Hispanic White participants. RSI is a surrogate marker of structural racism across census tracts. It is possible that all individuals living in census tracts with high RSI are affected by the poor neighborhood environments caused by structural racism-related discriminatory policies. In addition, we found stronger associations of NDI with all-cause and CVD mortality among women than men, which might be because women are, on average, economically disadvantaged compared with men and more likely to rely on neighborhood resources. Furthermore, we found that variations in neighborhood sociodemographic environment metrics and their associations with mortality could not be fully explained by standard SES and health risk factors, suggesting the involvement of other less-known factors. Given prior evidence that healthcare quality and access are impacted by sociodemographic ecology [61, 62], efforts to expand the reach of high-quality medical care to these sociodemographically disadvantaged communities may mitigate the residual mortality risk we observed, even after accounting for traditional SES measures.

Metabolic health and cell metabolism play essential roles in the development and progress of CVD and cancer [33, 63], and our study revealed potential circulating metabolites and metabolic pathways related to neighborhood sociodemographic environment. We found that higher NDI and RSI were associated with lower levels of metabolites in ascorbate/vitamin C and aldarate metabolism pathway (including threonate, 2-O-methylascorbic acid, ascorbic acid 3-sulfate, and ascorbic acid 2-sulfate) and xanthine/caffeine metabolism pathway (including caffeine, theobromine, paraxanthine, 1,7-dimethylurate, 3,7-dimethylurate, and theophylline), which may reflect lower consumptions of fresh vegetables, fruits, and coffee. In contrast, higher NDI was associated with higher levels of tobacco metabolites, including cotinine, hydroxycotinine, 3-hydroxycotinine glucuronide, and 2-hydroxyfluorene sulfate. Furthermore, higher NDI was associated with lower levels of many sphingomyelins. Reduced sphingomyelin levels have been linked to the risk of type 2 diabetes [64], and long-chain sphingomyelins were inversely associated with mortality [65]. Additionally, higher NDI was associated with higher levels of many metabolites in the urea cycle: arginine and proline metabolism pathway (including citrulline, n2,n5-diacetylornithine, trans-4-hydroxyproline, pro-hydroxy-pro, and 3-amino-2-piperidone), except lower level of homoarginine. Prior studies suggested that trans-4-hydroxyproline was a biomarker of processed meat consumption and associated with increased mortality [66, 67], while circulating homoarginine concentrations were inversely associated with mortality [68, 69]. Our metabolomics analysis unveiled potential pathways reflecting some of the effects of neighborhood sociodemographic environment on human metabolism, which may serve as biomarkers and help understand mechanisms linking social determinants with chronic disease development and mortality.

Our study complements current literature by assessing the associations of multiple neighborhood sociodemographic metrics with total and leading causes of death in a low-SES population in the US, including a large number of Black/African American adults, who have been underrepresented in existing studies while experiencing persistent socioeconomic challenges and health disparities. In addition, we presented the first study that explored the metabolite correlates of neighborhood sociodemographic environment and the mediation effects of circulating metabolites on the neighborhood environment-mortality association. Besides its novelty and inclusion of racially diverse, low-SES individuals, other strengths of our study include its prospective design, large general population-based cohort, multiple measures of neighborhood sociodemographic environment, and comprehensive metabolite profiling. However, we acknowledge several limitations in the current study. First, given the observational design, we cannot rule out residual confounding and make causal inferences from our findings. However, randomized social experiments have shown that reducing exposure to neighborhood poverty led to lower rates of extreme obesity and diabetes and potentially less hospitalization [70, 71]. Second, as NDI, RSI, and SVI were determined based on participants’ addresses at the time of cohort enrollment, we did not consider the change of address during follow-up nor the address before enrollment, which may introduce measurement error and potential bias to our findings. Third, high correlations between NDI, RSI, and SVI reflect the complex interplay in different aspects of the neighborhood environment, and we could not assess which aspect (neighborhood deprivation, residential segregation, or social vulnerability) is most associated with mortality. Finally, our identified neighborhood sociodemographic environment-related metabolites should be validated in other cohorts.

## Conclusions

Disadvantaged neighborhood sociodemographic environment (measured by NDI, RSI, and SVI) was associated with increased all-cause and CVD and cancer-specific mortality among low-income Black/African American adults and non-Hispanic White adults in the southeastern US, robust to comprehensive adjustment and consistent between racial groups. Standard clinical and sociodemographic risk factors only partially explained the variances in neighborhood sociodemographic metrics and their associations with mortality. Circulating metabolites may capture cumulative exposures to various measured and unmeasured factors related to neighborhood sociodemographic environment and uncover potential biological pathways for the impact of social determinants on human health. Our findings support public health efforts to reduce neighborhood-related sociodemographic and health disparities.

### Supplementary Information

Additional file 1: Figures S1–S3. Fig. S1 The associations of neighborhood sociodemographic environment metrics with all-cause and CVD and cancer-specific mortality by age. Fig. S2 The associations of neighborhood sociodemographic environment metrics with all-cause and CVD and cancer-specific mortality by sex. Fig. S3 Circulating metabolites associated with neighborhood sociodemographic environment metrics.

Additional file 2: Characteristics of participants included in the analysis of NDI/RSI/SVI and excluded due to missingness.

Additional file 3: The associations of neighborhood sociodemographic environment metrics with mortality among all participants and by self-reported race.

Additional file 4: The associations of neighborhood sociodemographic environment metrics with mortality by age (< median and ≥ median).

Additional file 5: The associations of neighborhood sociodemographic environment metrics with mortality by sex.

Additional file 6: The associations of neighborhood sociodemographic environment metrics with circulating metabolites.

Additional file 7: The result of pathway enrichment analysis for neighborhood sociodemographic environment-related metabolites.

## Data Availability

The data underlying this article can be obtained through the Southern Community Cohort Study (https://www.southerncommunitystudy.org/) upon reasonable request and approval by the SCCS Data and Biospecimen Use Committee.

## Abbreviations

BMI: Body mass index
CHD: Coronary heart disease
CI: Confidence interval
COPD: Chronic obstructive pulmonary disease
CVD: Cardiovascular disease
FDR: False discovery rate
HR: Hazard ratio
ICE: Index of concentration at the extremes
IQR: Interquartile range
NDI: Neighborhood deprivation index
RSI: Residential segregation index
SCCS: Southern community cohort study
SD: Standard deviation
SES: Socioeconomic status
SVI: Social vulnerability index
