# Supplementary material for: Associations of neighborhood sociodemographic environment with mortality and circulating metabolites among low-income black and white adults living in the southeastern United States
Source: BMC Med. 2024 Jun 18;22:249. doi: 10.1186/s12916-024-03452-6 (PMC11184804; doi:10.1186/s12916-024-03452-6)

# **Supplementary Materials**

# **Fig. S1. The associations of neighborhood sociodemographic environment metrics with all-cause and CVD and cancer-specific mortality by age (< median and ≥ median).** Cox regression and competing risk framework within Cox regression were used to examine the associations of NDI, RSI, and SVI with all-cause mortality and CVD and cancer-specific mortality, respectively. Model 1 included age, sex, and self-reported race; model 2 additionally included education, annual household income, and insurance coverage; model 3 additionally included smoking status, alcohol drinking, physical activity, diet quality, sleep hours, BMI, depression score, and histories of diabetes, hypertension, COPD, CVD, and cancer. Models were performed by age (< median and ≥ median). The median age was 51 years in our study. NDI, neighborhood deprivation index; RSI, residential segregation index; SVI, social vulnerability index; CVD, cardiovascular disease; HR, hazard ratio; CI, confidence interval; COPD, chronic obstructive pulmonary disease.


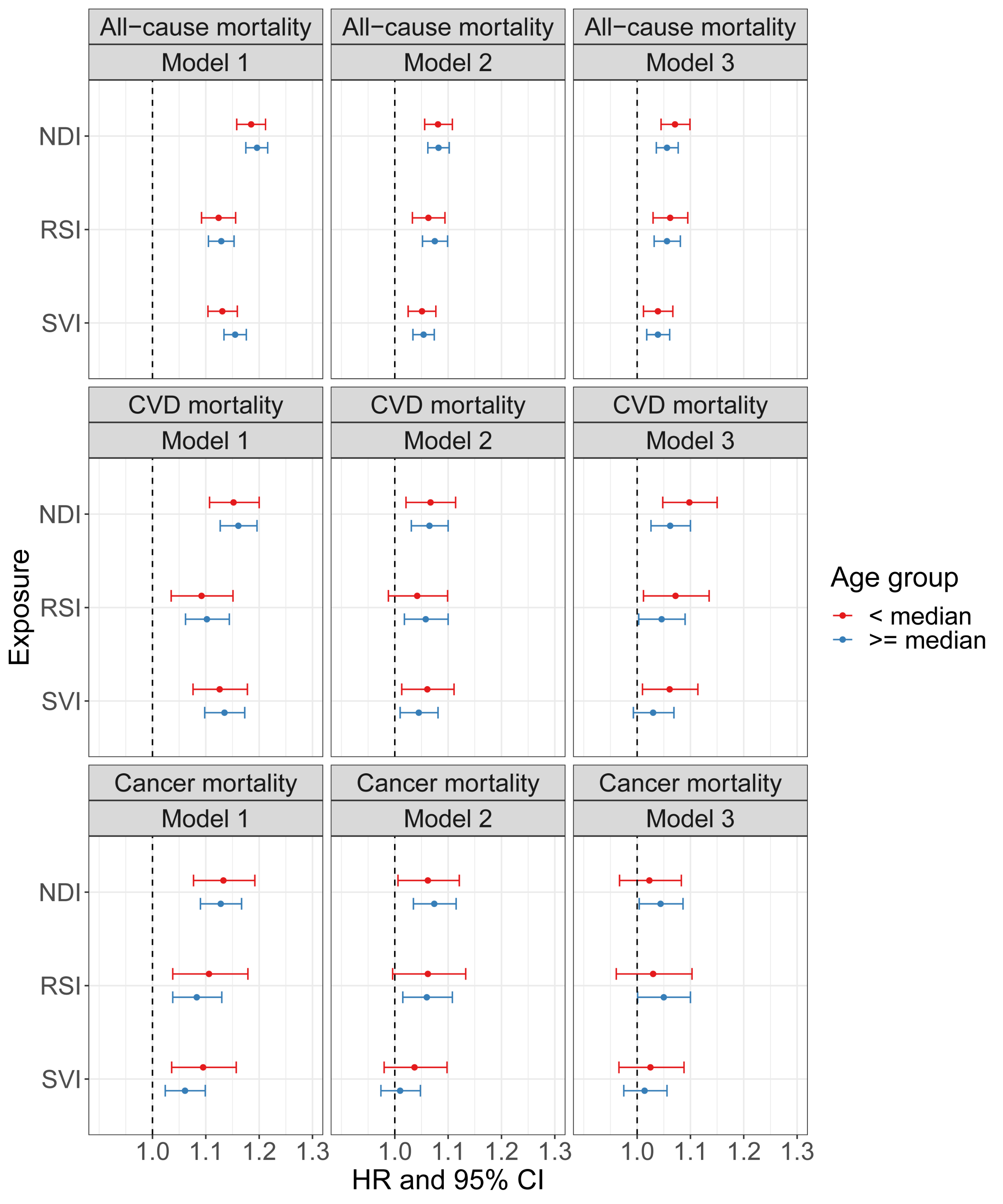


# **Fig. S2. The associations of neighborhood sociodemographic environment metrics with all-cause and CVD and cancer-specific mortality by sex.** Cox regression and competing risk framework within Cox regression were used to examine the associations of NDI, RSI, and SVI with all-cause mortality and CVD and cancer-specific mortality, respectively. Model 1 included age and self-reported race; model 2 additionally included education, annual household income, and insurance coverage; model 3 additionally included smoking status, alcohol drinking, physical activity, diet quality, sleep hours, BMI, depression score, and histories of diabetes, hypertension, COPD, CVD, and cancer. Models were performed by sex. NDI, neighborhood deprivation index; RSI, residential segregation index; SVI, social vulnerability index; CVD, cardiovascular disease; HR, hazard ratio; CI, confidence interval; COPD, chronic obstructive pulmonary disease.


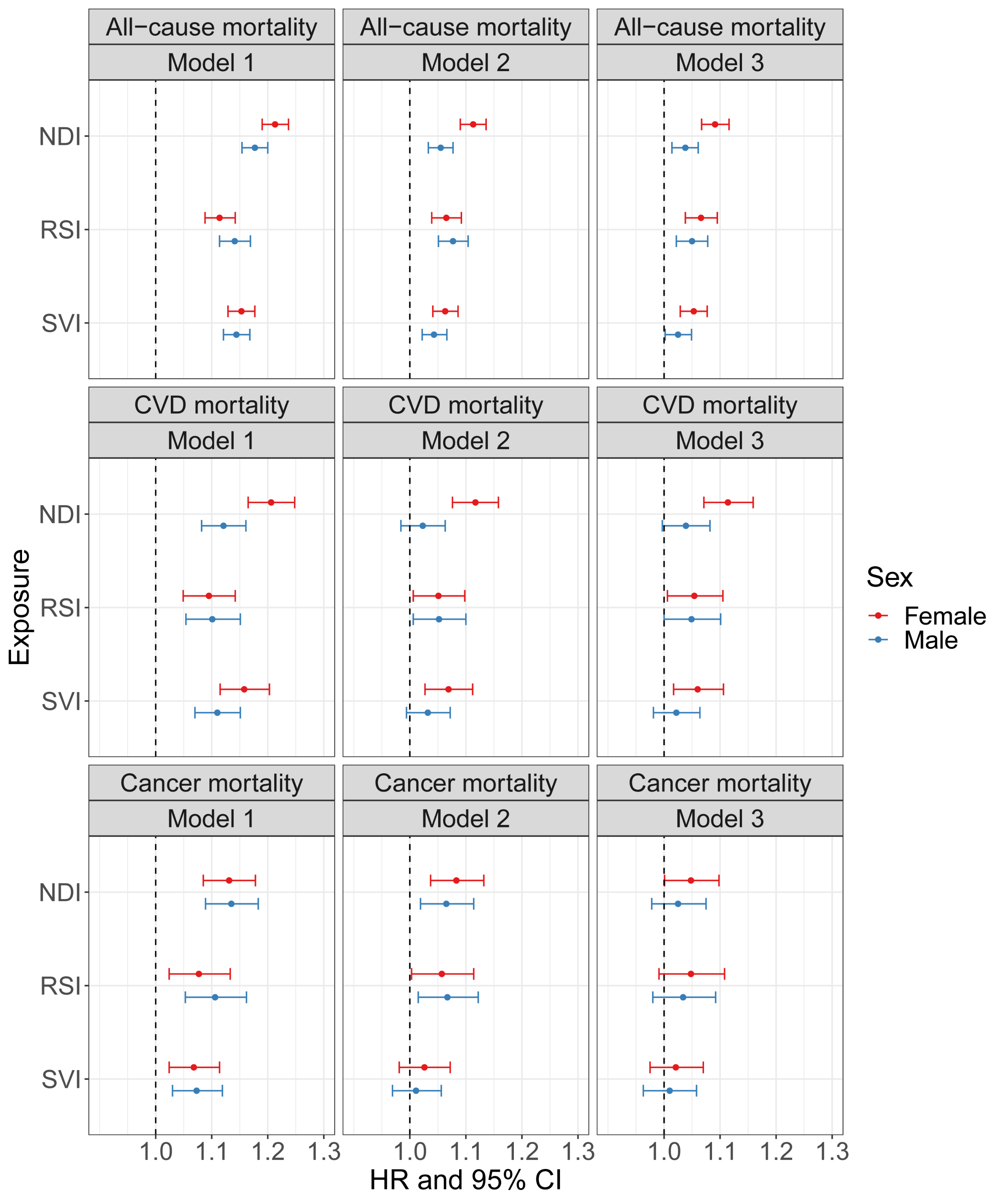


# **Fig. S3. Circulating metabolites associated with** **neighborhood sociodemographic environment metrics. (A)** Metabolites associated with neighborhood deprivation index. **(B)** Metabolites associated with residential segregation index. Metabolites with FDR<0.05 were highlighted in blue (negative associations) or red (positive associations). The top 10 significant metabolites were labeled.


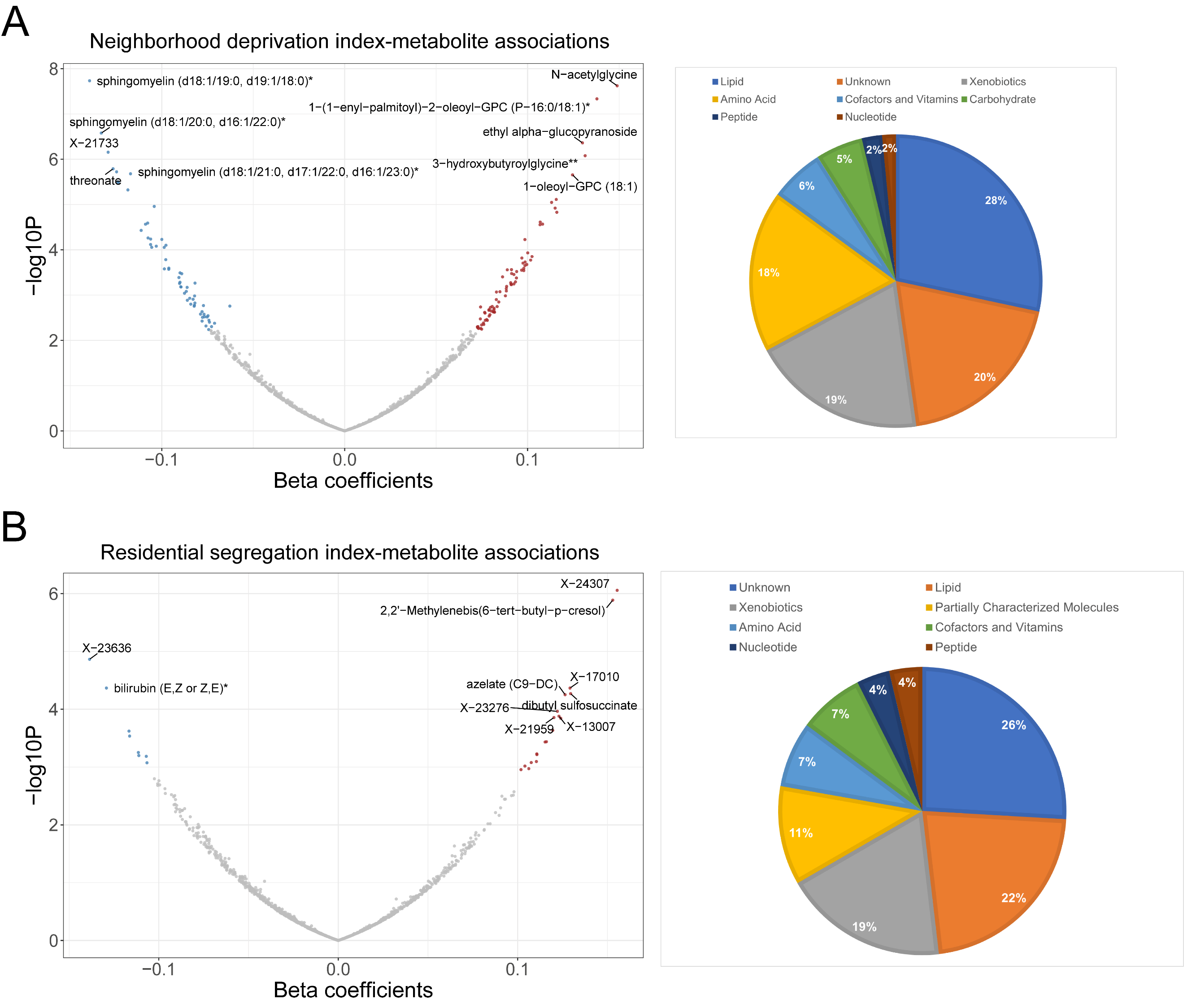

Supplement: Supplementary file 1 — Additional file 1: Figures S1–S3. Fig. S1 The associations of neighborhood sociodemographic environment metrics with all-cause and CVD and cancer-specific mortality by age. Fig. S2 The associations of neighborhood sociodemographic environment metrics with all-cause and CVD and cancer-specific mortality by sex. Fig. S3 Circulating metabolites associated with neighborhood sociodemographic environment metrics. [file 12916_2024_3452_MOESM1_ESM.docx]
